# Wax: A benign hydrogen-storage material that rapidly releases H_2_-rich gases through microwave-assisted catalytic decomposition

**DOI:** 10.1038/srep35315

**Published:** 2016-10-19

**Authors:** S. Gonzalez-Cortes, D. R. Slocombe, T. Xiao, A. Aldawsari, B. Yao, V. L. Kuznetsov, E. Liberti, A. I. Kirkland, M. S. Alkinani, H. A. Al-Megren, J. M. Thomas, P. P. Edwards

**Affiliations:** 1 King Abdulaziz City for Science and Technology Centre of Excellence in Petrochemicals, Inorganic Chemistry Laboratory, University of Oxford, South Parks Road, Oxford, OX1 3QR, UK; 2 School of Engineering, Cardiff University, Queen’s Buildings, The Parade, Cardiff, CF24 3AA, UK; 3 Department of Materials, University of Oxford, Holder Building, Parks Road, Oxford, OX1 3PH, UK; 4 Petrochemical Research Institute, King Abdulaziz City for Science and Technology, P. O. Box 6086, Riyadh 11442, Kingdom of Saudi Arabia; 5 Department of Materials Science and Metallurgy, University of Cambridge, 27 Charles Babbage Road, Cambridge, CB3 0FS, UK

## Abstract

Hydrogen is often described as the fuel of the future, especially for application in hydrogen powered fuel-cell vehicles (HFCV’s). However, its widespread implementation in this role has been thwarted by the lack of a lightweight, safe, on-board hydrogen storage material. Here we show that benign, readily-available hydrocarbon wax is capable of rapidly releasing large amounts of hydrogen through microwave-assisted catalytic decomposition. This discovery offers a new material and system for safe and efficient hydrogen storage and could facilitate its application in a HFCV. Importantly, hydrogen storage materials made of wax can be manufactured through completely sustainable processes utilizing biomass or other renewable feedstocks.

A hydrogen economy relates to the energetic, economic and environmental ramifications of using hydrogen, rather than electricity, as a primary vector of energy transport^1^,^2^,^3^,^4^,^5^. It is widely recognised that a major scientific and technological barrier to the commercialisation and market acceptance of hydrogen powered fuel cell vehicles (HFCV’s) is the lack of a cheap, safe and easily produced (onboard) hydrogen storage material with suitable hydrogen generation kinetics^6^,^7^,^8^,^9^,^10^,^11^,^12^,^13^,^14^,^15^. Despite strenuous efforts over the past decades covering a vast range of hydrogen storage materials (Fig. 1a), no single material has met simultaneously the critical requirements for a viable hydrogen storage and hydrogen releasing material suitable for use in HFCV’s and other fuel cell applications^6^,^7^,^8^,^9^,^10^,^11^,^12^,^13^.

In addition to these severe technical demands, the highest priority from a purely societal viewpoint is the need for a hydrogen storage material to be non-hazardous, non-toxic, inert (to air and water) and durable^6^,^10^,^11^,^13^. In addition, any viable hydrogen storage material should be amenable to large scale manufacture through environmentally-sustainable processes^6^,^7^. Chemical, complex and metallic hydrides all present significant difficulties of pyrophoricity, accidental hydrogen release (from accidental reaction with atmospheric moisture) and heat management problems, together with troublesome changes in particle morphology in the hydrogen charging/discharging processes^7^,^8^,^9^,^10^,^11^,^12^,^13^. Similarly, rechargeable organic liquids – used to store hydrogen in a liquid carrier form – must be handled with great care as several react violently with strong oxidants, have fire and explosion hazards and, in certain cases, carry toxicity concerns from occupational exposure^14^,^15^. Similar safety issues and concerns surround the potential use of ammonia as an on-board hydrogen storage material^16^.

Important efforts have been made previously to liberate pure hydrogen from the catalytic decomposition of the lightest alkane, methane^17^,^18^. Against this backdrop, we sought to investigate heavy alkane hydrocarbon waxes as cheap, safe, readily producible and widely accessible hydrogen storage materials. We reasoned that such materials – if suitably activated to rapidly release hydrogen, or hydrogen-rich mixtures – could exhibit many desirable features and yield hydrogen gravimetric densities approaching a theoretical value of ca. 14 wt% (Fig. 1a and inset). The greatest challenge is to devise a new way of dehydrogenating a hydrocarbon wax so as to release hydrogen effectively and rapidly, whilst minimising unwanted by-products. We chose a representative wax, C_26_H_54,_ and found that ca. 7 wt% hydrogen is rapidly produced (see Video in Supplementary Information) by microwave radiation-assisted catalysis involving ruthenium nanoparticles on a carbon support (CS), intimately dispersed within paraffin wax (PW).

As a preliminary, guiding principle, we carried out a detailed thermodynamic analysis of the so-called deep dehydrogenation process of alkane hydrocarbons to yield hydrogen and carbon, via Eq. (1):


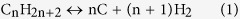


The variation in the computed Gibbs free energy (Δ_r_G) with both temperature and carbon nuclearity for deep hydrogenation reactions, using the *HSC chemistry 5* software^19^, is shown in Fig. 1b. These calculations clearly show that the deep dehydrogenation reactions necessary for efficient hydrogen formation reactions become more favourable, not only with increasing reaction temperature, but also with increasing number of carbon atoms in the lineal alkane. This trend is driven partly by the increasingly favourable entropic contribution (TΔS) with increasing carbon nuclearity.

This initial thermodynamic analysis highlighted heavy hydrocarbon paraffin waxes as potential hydrogen production and storage materials. However, conventional thermal catalysis of hydrocarbon waxes yields a variety of cracking products, mainly lighter hydrocarbons^20^. In a related area, our studies of microwave absorption in small conducting particles^21^, when dispersed in heavy bunker oils, similar chemically to waxes, revealed the considerable potential of microwave heating of metallic, catalytic particles for the generation of hydrogen gas^22^ as compared to conventional thermal catalytic processes^23^.

The experimental setup (Fig. 2) consisted of a microwave generation and control system, a purpose-built microwave cavity and associated on-line gas chromatography (GC) apparatus. Figure 2 also shows both finite element model representations of the TM_010_ electric field distribution in the resonant microwave cavity and the electric field energy density in the catalyst bed (Fig. 2a), together with a schematic representation of the potential formation of hotspots at the interface between the paraffin wax and the metal catalyst during microwave irradiation (Fig. 2b).

A large number of catalysts were dispersed in PW and supported on AC’s and subjected to controlled microwave irradiation. Resulting gas and liquid samples were collected at various times during the microwave irradiation of the samples and analysed by GC. Microwave electric field activation was carried out in a single-mode resonant cavity with minimal mechanical tuning and hence the absorbed power was inferred by subtracting the measured reflected power from the forward (input) power generated by the magnetron based system, assuming negligible radiation and cavity wall losses.

The results of gas evolution experiments (see Video Fig. S1) using microwave activation of carbon-supported ruthenium catalyst-loaded samples are compared with those of ruthenium-catalyst-free systems in “time-on-stream” experiments in Fig. 3. This series of experiments was carried out by keeping the input microwave power constant (i.e. 500 W for Fig. 3a,b and 100 W for Fig. 3c) whilst the (monitored) absorbed microwave power varied from 80 to 60 W (Fig. 3a,b) and from 20 to 15 W (Fig. 3c) with reaction time. The gaseous products were analysed at different microwave reaction times or “time-on-stream”. In this report we concentrate on the use of ruthenium catalytic particles.

The hydrogen yield for samples containing only paraffin waxes dispersed on AC was low and the produced hydrogen volume did not vary markedly with microwave reaction time (Fig. 3a). The relatively large co-production of light hydrocarbons (C_2_–C_5_) and particularly olefins (C_2_–C_4_^=^) indicate that the established radical mechanism appears to operate under these reaction conditions^24^.

In stark contrast, the hydrogen concentration (80–60 mol.%), obtained in the presence of the ruthenium metal catalyst (Fig. 3b) is significantly larger than that obtained (ca. 40 mol.%) for microwave irradiation in the absence of the metal catalyst. The ruthenium metal catalyst/paraffin wax samples on CS (e.g. @5 wt.% Ru/AC) show rapid hydrogen production from the onset of the microwave assisted catalytic decomposition of the wax (even at ca. 30 s). This is presumably as a consequence of the large availability of surfaces of activated ruthenium metal nanoparticles. This rapid hydrogen formation also coincides with the maximum level of hydrogen produced. For the ruthenium catalyst samples studied, the hydrogen concentration decreased after ca. 1 min. of time-on-stream, whilst the corresponding methane concentration gradually increased, indicative of catalyst deactivation by evolving carbon deposition and/or the coalescence of the ruthenium nanoparticles, both of which could promote secondary reaction pathways that facilitate the scission of C–C rather than C–H bonds. A lower absorbed power (20–15 W) in the paraffin waxes @ 5 wt.% Ru/AC composite did not markedly affect the gas composition (Fig. 3c) but required a longer reaction time to release an equivalent amount of hydrogen. Figure 3d illustrates the substantial increase in the hydrogen production rate, compared to that of methane and C_2_–C_5_ compounds, with increased absorbed microwave power. Each experiment was carried out at a constant input power of 100, 300, 500 and 750 W and the absorbed power and gas yields were determined with time-on-stream. Fresh samples were used for each experiment with the amount of sample kept approximately constant, typically, between 0.80 and 0.82 g. The catalyst-bed surface temperature (monitored directly by an IR pyrometer, Fig. 2a) initially increased up to 743 K and then slowly decreased to ca. 673 K during the reaction time for adsorbed microwave powers of ca. 30, 60 and 115 W. We attribute this behaviour to the partial coverage of the surface of metal particles by carbon produced following the hydrogen release process. The hydrogen formation rates are clearly higher than those associated with methane production in the presence, or indeed in the absence, of a metal catalyst power (see Supplementary Fig. S1) and over wide ranges of the absorbed microwave (Fig. 3d).

The final resulting distribution of gaseous products is shown in the “Spider plot” in Fig. 3e. The use of activated carbon black as the only microwave absorber dispersed in paraffin wax efficiently facilitates the formation of light alkenes (C_2_^=^–C_4_^=^), most likely through thermal cracking via a radical mechanism^24^. In contrast, the overall production of hydrogen from the decomposition of waxes over ruthenium, platinum and palladium catalysts showed a significantly higher production of hydrogen than those produced from the decomposition of only carbon-paraffin wax materials. The characterization of fresh and spent activated carbon and ruthenium-containing samples by both Thermal Gravimetric Analysis and Differential Scanning Calorimetry clearly highlights the positive effect of the metal catalyst in the decomposition of the wax (see Supplementary Data Fig. S2 and Supplementary Table S1).

We find that the presence of a ruthenium (or platinum or palladium) metal catalyst gives a significant increase in the amount of hydrogen produced, together with a decrease in the formation of C_2_–C_5_ hydrocarbons and light olefins (C_2_–C_4_^=^) concentrations. A preliminary comparison of the catalytic performances for carbon-dispersed ruthenium, palladium and platinum catalysts is presented in Supplementary Table S2.

Crucially, around half of the maximum (theoretical) gravimetric hydrogen content (14.8 wt.%) of the paraffin wax can be released by using noble metal catalysts (palladium, platinum and ruthenium) and microwave radiation in the catalytic decomposition process of the paraffin wax (Supplementary Table S2). We believe that this yield can be enhanced further by optimizing the catalyst composition and the reactor-microwave configuration using an appropriate engineered design^25^ and investigations of alternative metal catalysts, e.g. transition metals. We also compared, in detail, the effect of heating sources (i.e. microwave radiation versus conventional thermal heating) in the catalytic decomposition of this wax (Supplementary Table S3). From these studies, it is clear that using microwave radiation as a heating source enhances not only paraffin wax conversion, but also hydrogen production (a summary is presented in Supplementary Fig. S3) as compared to conventionally thermal -heated samples.

The ruthenium catalyst particles were examined before and after microwave treatment by aberration corrected High-Resolution Transmission Electron Microscopy (HRTEM). A representative HRTEM phase contrast image (recorded under negative Cs imaging conditions) of a ruthenium catalyst nanoparticle on activated carbon containing paraffin wax (prior to microwave activation) is shown in Fig. 4a. For ruthenium samples on AC (Supplementary Fig. S4A,B), the particles had a hexagonal closed packed crystal structure^26^, with average diameters of ca. 3 nm.

Following microwave treatment, the ruthenium catalyst maintains its shape and crystal structure (Fig. 4b). The average diameter of the catalyst particles after treatment is generally slightly larger than the pristine catalyst (<5 nm). This increase in the particle size is due to Ostwald ripening and sintering during the microwave-assisted catalytic reaction^27^.

Despite the high content of carbon and carbonaceous material produced in the microwave-assisted deep dehydrogenation of paraffin wax, the ruthenium catalyst nanoparticles retained their hcp crystal structure. Analysis of the power spectra, calculated from the images obtained from Fig. 4a,b, showed no evidence for the formation of any ruthenium carbide phase^28^.

Previous studies have described the microwave-assisted production of hydrogen from light hydrocarbons, e.g. methane^17^, as well as the high temperature catalytic decomposition of gasoline-range alkanes, with reduced CO_2_ emissions^29^. Following the proposal of Otsuka *et al*. for the reaction pathways for hydrogen production from gasoline-range alkanes^29^ the catalytic decomposition for heavier alkane wax may be similarly conceptualised by considering the competing processes of cleavage of the constituent C–H and C–C bonds. The scission of the C–H bonds through the partial dehydrogenation to produce alkenes and alkynes and deep dehydrogenation to generate residual carbon could take place through a consecutive mechanism that releases hydrogen in each step of this reaction network (see Eqs (2, 3, 4)).


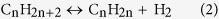



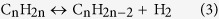



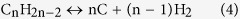


On the other hand, the cleavage of terminal (Eqs (5) and (6)) and/or intermediate C–C bonds (Eqs (7) and (8)) for i ≥ 2 and n > i) through cracking and hydrogenolysis-type secondary reactions of *n*-alkanes can produce methane and other small molecular fragments (saturated and/or unsaturated compounds), which are illustrated in the below set of chemical reactions (Eqs (5, 6, 7, 8)). All these reaction pathways may occur upon the microwave-assisted catalytic decomposition of wax, the hydrogen generation through the scission of the C–H bonds appears to be mainly catalyzed by metal catalyst whilst the production of light hydrocarbons (C_1_–C_4_), particularly methane, through the cleavage of C–C bonds enhanced by carbon material. However, we cannot rule out the hydrogenation reaction of residual carbon to produce methane.


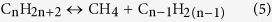



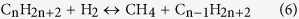



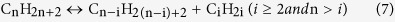



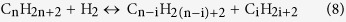


Crucially, microwave heating of heterogene.ous catalyst mixtures is fundamentally different from conventional heating in two important aspects^30^,^31^.

*Firstly*; the heat is generated selectively throughout the sample in regions of high microwave absorption, as opposed to conventional thermal (bulk) heating, where heat is transferred to the sample through the system boundaries. Dramatically redistributed fields within heterogeneous mixtures can cause the formation of “hotspots” (Fig. 2)^30^,^31^. However, unlike hotspots found in conventional thermal catalysis, for microwave-assisted catalysis these are driven by very high electric fields (Fig. 2a) and can lead, amongst several effects, to highly localised superheating at the surface of the catalyst. The occurrence of this phenomenon in the interface between the metal catalyst and the paraffin wax will induce significant temperature gradients between the catalyst particle hotspot and the surrounding domains. We believe that this non-equilibrium localised superheating of the metal catalyst particle speeds up the release of hydrogen through catalytic decomposition of the wax in a deep dehydrogenation reaction (Fig. 2b). In contrast, the formation of small amounts of light gaseous products (i.e. C_1_–C_4_), particularly methane, is likely associated with a thermal cracking reaction of paraffin wax. Although our IR pyrometry showed that during the reaction the temperature of the sample was around 573–743 K, such highly localised hotspots would not be detected by the pyrometer and are thus expected to be at much higher temperatures.

*Secondly;* the high electric fields associated with the surface polarisation of small metallic catalytic particles will also dramatically enhance any dielectric heating for species in the immediate vicinity (interface) of the particle surface, such that plasma generation and field ionisation could also contribute to the decomposition processes (Fig. 2).

The extent to which the observed rapid hydrogen production depends on electromagnetically generated hotspots in the catalyst alone, or whether high-field effects also contribute, has yet to be determined. Although the precise details of the observed catalytic decomposition of the wax remain to be fully explored, it is clear that microwave irradiation, in the presence of metal catalyst particles, appears to favour the preferential rupture of C–H rather than C–C bonds, possibly due to differences in the C–X (X=H, C) bond polarizability. These findings once again highlight the unique heat transfer characteristics of microwave heating, derived from the selective or differential heating of a strongly microwave absorbing component. We anticipate that at the metal particle surfaces (the location of the catalytic sites) surface field enhancements may lead to superheating at those sites.

These findings offer a different approach to the oft-cited “grand challenge” for hydrogen storage materials^13^. Clearly, more studies are needed to span the entire innovation cycle from small-scale laboratory hydrogen production to industrial-level processes. A complete well-to-wheel life cycle analysis for the entire hydrogen production process will highlight the translational and applied topics, but at this stage it is already possible to identify several key areas.

The efficiency of hydrogen production can be evaluated by the “Net energy balance “(NEB) ratio which simply gives the ratio of chemical or electrochemical energy derived from the products (energy out) to the energy invested (energy in) in the process^32^. For simplicity, equating the energy out as the enthalpy of combustion of (only) the hydrogen produced, and the energy in as the absorbed microwave power, at present the net energy balance is very low (ca 1% efficiency or below). Such poor efficiencies are often observed when a high power microwave device is used for small sample volumes and it is recognised that more effective utilisation of microwave power (e.g. by impedance matching and integrated feedback systems) can lead to significant process energy savings. Furthermore, recent advances in the area of solid-state microwave power amplifiers show that compact, highly efficient microwave sources can be used for on-board generation of hydrogen in a suitably small device^33^.

We highlight also the high yield and speed of hydrogen production under microwave irradiation, both aspects being key for any adoption in automotive application.

Under microwave irradiation conditions, hydrogen is the predominant product (Fig. 3) with smaller quantities of CH_4_ and higher molecular weight hydrocarbons (e.g. C_2_–C_5_). For any subsequent hydrogen separation and purification, membrane separation is recognised as an attractive route because of its ease of operation, low energy consumption and cost effectiveness^34^. Furthermore, a low temperature proton exchange membrane (PEM) fuel cell can also be used as a separating device to obtain pure hydrogen from a mixture with CH_4_, the latter being essentially inert at the low operating temperatures of the fuel cell^35^. Another option is the process enhancement of hydrogen production and equivalent reduction of light hydrocarbon production by catalyst formulation and optimisation.

One of the most effective microwave-active catalyst we have found to-date is ruthenium and although it is the least expensive of the precious group metals ($42/troy ounce as compared to $854 for Pt) further work on reducing the amount of ruthenium and/or/alloying with other metals needs investigation. Regeneration tests revealed that the ruthenium catalyst is still active after the first cycle, providing 56% of the hydrogen yield produced in the first catalytic cycle (7.9 wt.%); a third catalytic cycle delivered 37% of the hydrogen yield relative to the first catalyst run. This trend is attributed to the poisoning of the active ruthenium in the re-used catalyst by carbonaceous materials on the catalyst surface. This further supports ruthenium as the active component for the microwave assisted process and we are currently developing a fixed bed catalyst structure to reduce the effect of carbon residues on catalyst performance.

If the high performance levels of hydrogen production could be maintained or even improved, bi-metallic ruthenium-iron nanoparticles would enable magnetic recovery of the catalysts^36^. Further experiments are ongoing to evaluate base metals such as iron and cobalt as earth-abundant catalysts, to reflect potential usage for scale up processes. Further maximization of the hydrogen yield can be targeted by studies of such alternative metal catalysts and/or by varying the microwave parameters and optimization of the reaction.

Finally, when separated from the catalyst, the resulting carbon residue can be either hydrogenated, utilising renewable hydrogen, or gasified into syngas, which can be subsequently recycled into waxes through the established Fischer Tropsch process^37^,^38^,^39^.

The research described here presents a new combined microwave-activated catalytic process for the rapid release of hydrogen, sufficient to meet the 7 wt% target set by the US DOE^13^. Clearly, considerable engineering work is needed to adapt this laboratory discovery to large-scale hydrogen storage applications. However, we believe that the storage of hydrogen in, and rapid evolution from, paraffin wax could usher in a new and attractive path towards a decarbonized, hydrogen economy, as illustrated in Fig. 5.

Wax is the major product of the low temperature Fischer-Tropsch synthesis process from syngas^37^,^38^,^39^ and is currently thermally “cracked” to produce various fuels. Importantly, it can therefore also be manufactured using renewable energy and any carbon-containing resources including biomass, CO_2_, natural gas, coal and, of course, the resulting carbon residue from the hydrogen-depleted wax (Fig. 5). Overall, this advance can help define a roadmap for a sustainable energy future through the decarbonisation of mobile applications^6^.

## Methods

### Synthesis of Ru catalyst

We synthesised 5.00 g of carbon-supported 5 wt.% Ru catalyst. Ru (II) nitrosyl nitrate solution (16.67 g) (Sigma-Aldrich, 1.5% Ru) was slowly added to 4.75 g of activated carbon (Norit) dispersed in deionised water (55 cm^3^/support-g), the mixture was vigorously stirred at 333 K for 4 hours under reflux. The mixture was left to cool to room temperature overnight and its pH was adjusted to between 9 and 10 units using sodium hydrogen carbonate (0.90–1.10 g) (99%). Subsequently, 50 g of diethylene glycol (Sigma-Aldrich, 99%) was added to the solution under vigorous stirring for a period of 10–15 minutes and heated under reflux to 433 K for 4 h. The solution was then cooled again to room temperature and the carbon-supported Ru catalyst was separated from the aqueous solution by filtration. The resultant solid was extensively washed with 2.5 L of deionised water until the filtrate solution achieved neutral pH. The solid was finally dried at 363 K for 16 h and reduced in a 10% H_2_-Ar mixture at 723 K for 4 h using a heating rate of 0.083 K.s^−1^ and a weight time space velocity of 1.67 mL(g.s)^−1^.

### Synthesis of the blend of paraffin waxes and catalyst

We prepared between 2.50 and 5.00 g of blends (composites) of paraffin waxes (PW) and catalceptor (i.e. material able to absorb microwave radiation and catalyse the decomposition of the substrate) into a glove box under a nitrogen (>99.9%) atmosphere with silica gel as a moisture adsorbent. As an example to synthesize 2.50 g of composite containing 50 wt.% of PW, we initially weighed 1.25 g of paraffin waxes (m.p. 326–330 K, Sigma-Aldrich) in a 100 mL beaker and melted in a hot plate located into the glove box. Subsequently, 1.25 g of the catalceptor such as commercially available activated carbon (Norit), carbon black (Sigma-Aldrich), activated carbon-supported noble metal (Pt, Pd, Ru) catalysts (Sigma-Aldrich) or synthesized, activated carbon-supported Ru catalyst previously reduced in 10% H_2_-Ar mixture was slowly added to the melted paraffin wax. This process was carried out with continuous and gentle stirring of the slurry for 20 minutes to obtain a homogenous solid mixture after cooling to room temperature. In order to obtain uniform particle sizes in the composite (<500 μm), the slurry was continuously during the cooling process until the sample reached room temperature. The composites were subsequently sored in an oxygen- and moisture-free atmosphere to preserve the identity of the metal catalyst.

### Catalytic tests under microwave irradiation

The microwave heating system chain prior to the applicator consisted of a power generator, microwave head, microwave circulator, dummy load, microwave power meters and tuneable waveguide sections (Sairem Ltd.). The system was computer controlled using the Labview software. The applicator section was fabricated in the Inorganic Chemistry Laboratory at the University of Oxford. The operating frequency was 2450 MHz (±25 MHz) from 10% to 100% of nominal power. The maximum output power was 2000 W with 1% stability from 10% to 100% of maximum power after thirty minutes on. The microwave source is a magnetron with a ripple rate of <1% RMS from 10% to 100%. The power rise time is about 100 μs. The power generator is a resonant switching converter with frequencies of 30 kHz up to 80 kHz and an efficiency of 93% at nominal power. The power supply and microwave head are both water and air cooled. The microwave output is via WR 340 waveguides. The generator is controlled remotely via an RS232 MODBUS gateway using the Labview software. The reflected power *R*, was measured by a crystal detector mounted onto the isolator load, from which we determine the power absorbed by the sample, *P*. If the power transmitted to the applicator is *W*, then *P* = *W* − *R* − *X*, where *X* is the microwave power lost in the cavity walls and by minor applicator leakages. The applicator used in this work is a TM_010_ resonant cavity to enable a well-characterized field distribution and high nominal field strength. Thus, in this work we investigate electric field driven processes, with a high electric energy density in the sample region.

We measured the sample temperature using an infrared (IR) pyrometer, which was also used to control the power to the generator. The pyrometer was positioned horizontally to face a side hole in the microwave cavity. The IR thermometer can only measure the external surface temperature of the catalyst. During the microwave experiment a temperature (T) versus time (t) of reaction profile was recorded. Typically, the power that was delivered to the sample by the microwave radiation and which is dissipated over the sample volume was between 20 W and 200 W.

**Figure 2:**
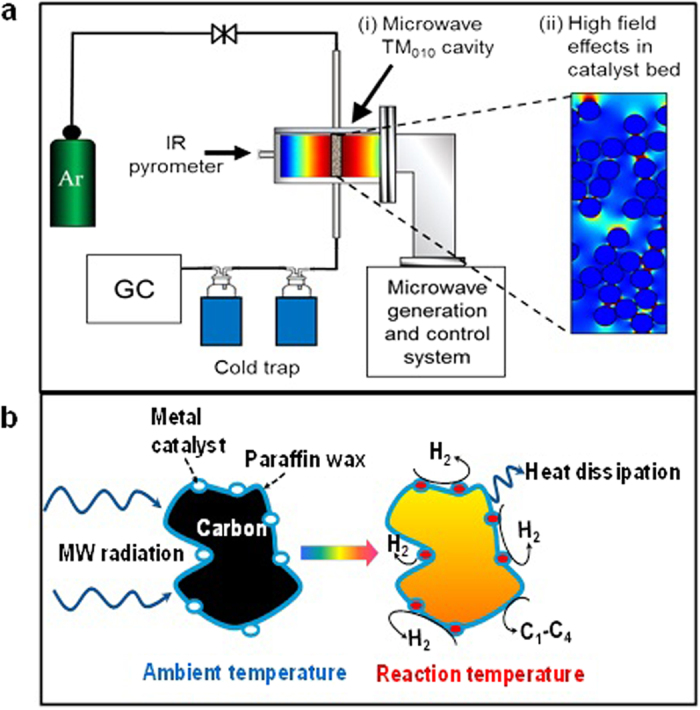
Experimental Apparatus. (**a**) Schematic representation of the experimental apparatus together with results of finite element models of (i) the TM_010_ electric field distribution in the resonant microwave cavity, showing the highly uniform, axially polarised antinode in the region of the catalyst bed (high field in red), and (ii) the electric field energy density in the catalyst bed during microwave irradiation: Electric field enhancement at particle surfaces is clearly visible (high field in red, low field energy in blue), which could have a dramatic influence upon the local catalyst environment. Note also a synergistic, cooperative effect between neighbouring catalyst particles. (**b**) Pictorial representation of the hydrogen evolution reaction from the microwave-assisted catalytic decomposition of paraffin wax on carbon-supported metal catalyst.

The sample volume was typically about 3.5 cm^3^. The sample was exposed to the microwave radiation until no more gas generation occurred, which corresponded to a maximum period of 90 minutes. In a typical procedure, approximately 0.80 g of the composite was loaded into the middle zone of the quartz tube reactor (22 cm in length and 1 cm in inner diameter) between quartz wool plugs at both side of the fixed-bed composite. Subsequently, we rapidly located the quartz tube reactor, sealed in nitrogen atmosphere, into the microwave cavity and connected to the inert carrier gas pipeline and immediately purged with an argon flow rate of 1.67 mL · s^−1^ for a period of 15 minutes (see experimental setup in Fig. 2).

In order to establish the effect of the microwave (absorbed) power on the thermal and catalytic decomposition of paraffin waxes, a series of experiments was carried out, keeping constant the input power and the irradiation time and collecting gaseous samples at different reaction times. The gas products were analysed by gas chromatography using a Perkin-Elmer, Clarus 580 GC.

In another series of experiments, the composite was irradiated with an absorbed power of 20–15 W for nearly 30 s, and the absorbed power was then increased to 30 W and held for another 30 s. Subsequently, the power was furher increased to 60 W and held for 60 s. Finally, the absorbed power was increased to 170–180 W and held for a long period (20–35 min) until no further gas generation was produced.

We also assessed the influence of the heating source (microwave radiation versus conventional thermal treatment). The composite-loaded reactor was purged with a nitrogen flow rate of 1.67 mL · s^−1^ for a period of 15 minutes; the reactor was then introduced into the furnace at 823 K and held for 20 minutes. The microwave experiment, on the other hand, was carried out at 80 W for 30 minutes reaction time. The gas products were analysed by gas chromatography using a Perkin-Elmer, Clarus 580 GC.

### High-resolution transmission electron microscopy (HRTEM)

High-resolution transmission electron microscopy (HRTEM) was carried out in a double aberration-corrected JEOL 2200MCO instrument operated at 200 kV^40^. For the 58 wt.% of paraffin wax @ 5 wt.% Ru/AC before microwave-assisted catalytic reaction, washing in toluene was first conducted to avoid hydrocarbon contamination during imaging. This process was carried out using 0.5 g of the sample and 80 mL of anhydrous toluene (Sigma-Aldrich, 99.8%) at 353 K for 4 h. The solid sample was separated from toluene by filtration and dried at 373 K for 12 h. Subsequently, the catalyst powder was dispersed in ethanol. The solution was then bath sonicated for 15 min, and drop cast onto a 300-mesh copper TEM holey carbon grid, on a filter paper and allowed to evaporate.

### Differential scanning calorimetry (DSC) and thermogravimetric analysis (TGA)

Simultaneous differential scanning calorimetry (DSC) and thermogravimetric analysis (TGA) were carried out using a SDT (Simultaneous DSC-TGA) Q600 instrument. TGA-DSC profiles were recorded from room temperature to 1273 K, using a 100 cm^3^ min^−1^ dry and oxygen-free nitrogen flow (99.99%) and 0.17 K s^−1^ heating rate. For each analysis between 10 and 20 mg of sample was loaded into a small alumina crucible, using alumina as reference.

## Additional Information

**How to cite this article**: Gonzalez-Cortes, S. *et al*. Wax: A benign hydrogen-storage material that rapidly releases H_2_-rich gases through microwave-assisted catalytic decomposition. *Sci. Rep.* **6**, 35315; doi: 10.1038/srep35315 (2016).

## Supplementary Material

Supplementary Information

## Figures and Tables

**Figure 1:**
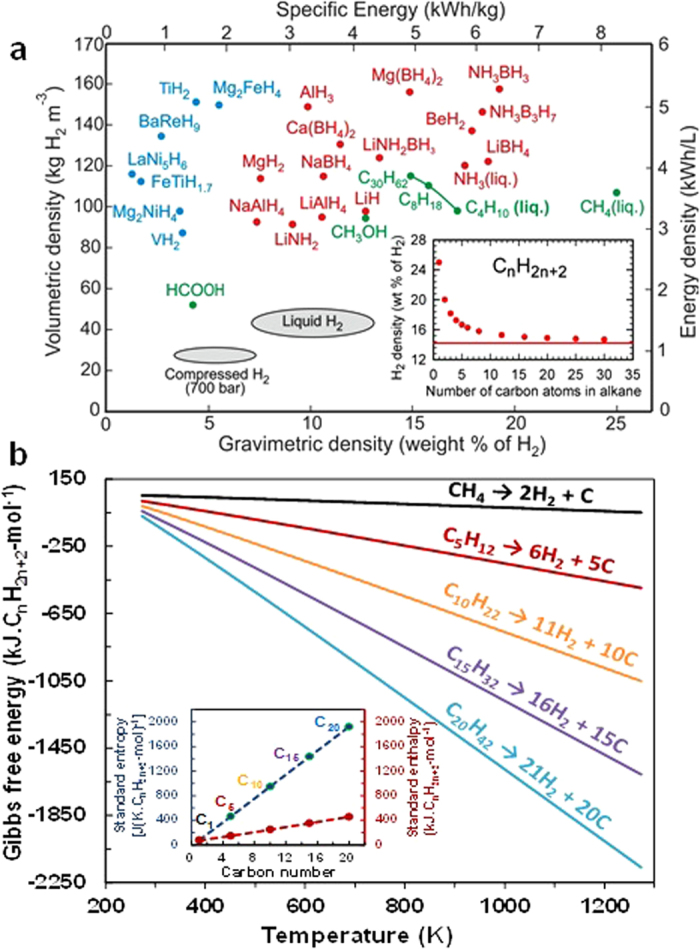
Alternative Hydrogen Storage Materials. (**a**) Gravimetric and volumetric densities of various hydrogen storage materials and options. Inset shows the hydrogen storage density of alkanes as a function of the number of carbon atoms. (**b**) Dependence of the Gibbs free energy with temperature for the deep dehydrogenation reaction (or hydrogen formation reactions) of various straight-chain alkanes (i.e. CH_4_; *n*-C_5_H_12_, *n*-C_10_H_22_, *n*-C_15_H_32_ and *n*-C_20_H_42_). Inset shows the dependence of the standard enthalpy and entropy for the hydrogen formation reactions with carbon numbers of various lineal alkanes.

**Figure 3:**
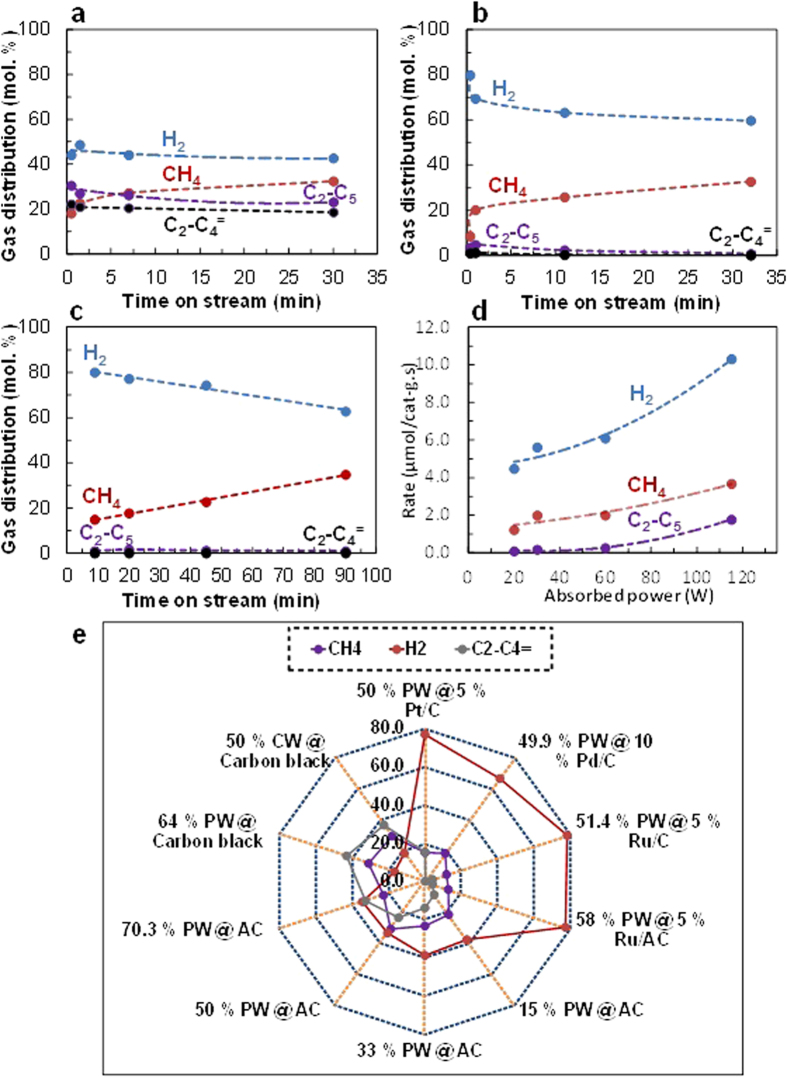
Evolved gas compositions with time-on-stream and absorbed microwave power. (**a**) 45 wt.% PW @ AC for absorbed power between 80 and 60 W. (**b**) 35 wt.% PW @ 5 wt.% Ru/AC for absorbed power between 80 and 60 W. (**c**) 35 wt.% PW @ 5 wt.% Ru/AC for absorbed power between 20 and −15 W. (**d**) The effect of absorbed power on the reaction rate of hydrogen, methane and C_2_–C_5_ production. Reactions conducted over 35 wt.% PW @ 5 wt.% Ru/AC for a period between 20 and 30 minutes. (**e**) Evolved gas distributions (mol%) for various catalysts assessed at atmospheric pressure.

**Figure 4:**
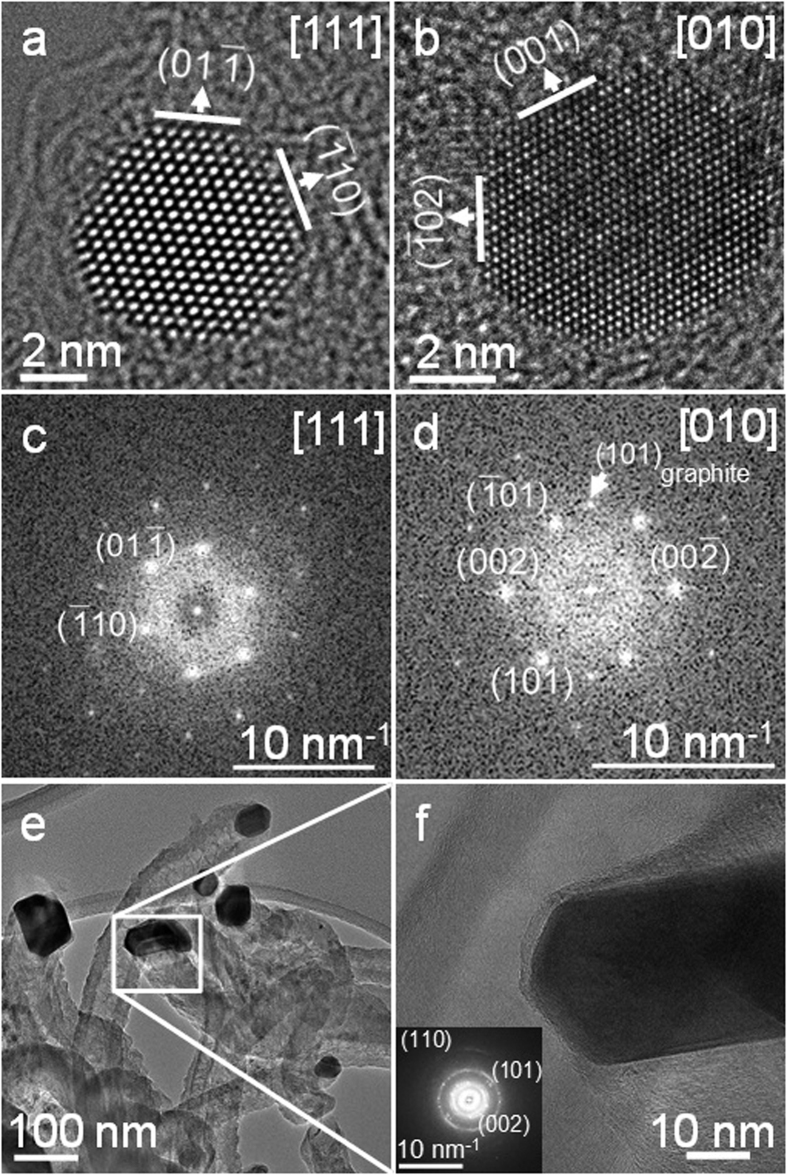
Representative HRTEM images of 58 wt.% PW @ 5 wt.% Ru/AC. (**a**) Before and (**b**) after images recorded along a <010> zone axis with {001} and {102} terminating facets following microwave-assisted catalytic decomposition of PW. Line annotations indicate terminating crystal planes of the hcp ruthenium nanoparticle and their normals (arrows). (**c**) Power spectra calculated from images of Ru particles of 58 wt.% PW @ 5 wt.% Ru/AC, before; and (**d**) after microwave-assisted catalytic decomposition of paraffin wax, showing characteristic reflections from ruthenium metal, along <111> and <010> zone axes, respectively. In (**d**) the arrow indicates the (101) planes of graphitic carbon. (**e**) Low magnification TEM images of ruthenium particles in a sample of 58 wt.% PW @ 5 wt.% Ru/AC after microwave-assisted catalytic reaction. (**f**) High-resolution image showing a graphite-like nanotube that encapsulates the catalyst. The power spectrum inset shows only graphite reflections. Following the microwave-assisted catalytic decomposition of paraffin wax, ruthenium catalyst particles of larger sizes with an average diameter of some tens-of-nanometres were occasionally observed. An example is shown in Fig. 4e (see also Table S4 for further analytical data). These particles were often embedded in a graphite-like carbon nanotube. The presence of graphite is clearly indicated in the power spectrum of Fig. 4f.

**Figure 5:**
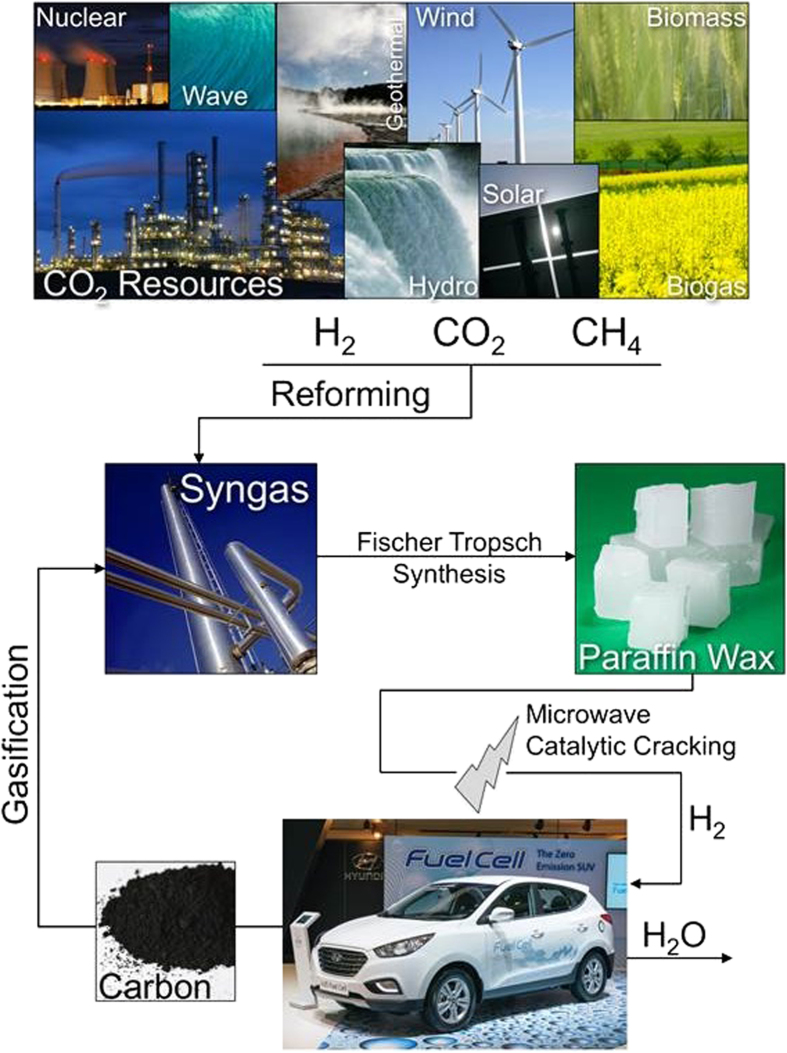
A Carbon Free Transportation Fuel Economy. Schematic representation of one scenario for the decarbonisation of a transportation fuel economy.
