# Supplementary material for: Wax: A benign hydrogen-storage material that rapidly releases H2-rich gases through microwave-assisted catalytic decomposition
Source: Sci Rep. 2016 Oct 19;6:35315. doi: 10.1038/srep35315 (PMC5069496; doi:10.1038/srep35315)
Supplement: Supplementary Information [file srep35315-s1.docx]

Supplementary information

Wax: A benign hydrogen-storage material that rapidly releases H_2_-rich gases through microwave-assisted catalytic decomposition

Sergio Gonzalez-Cortes, Daniel R. Slocombe, Tiancun Xiao, Afrah Aldawsari, Benzhen Yao, Vladimir L. Kuznetsov, Emanuela Liberti, Angus Kirkland, Mohammed S. Alkinani, Hamid A. Al-Megren, John Meurig Thomas and Peter P. Edwards

Supplementary Fig. S1

Supplementary Fig. S2

Supplementary Fig. S3

Supplementary Fig. S4

Supplementary Table S1

Supplementary Table S2

Supplementary Table S3

Supplementary Table S4

**Supplementary Fig. S1**

**Dependence of the hydrogen and methane formation reaction rates with the reaction time. a,** 45 wt. % paraffin wax @ activated carbon at absorbed power of 80 W for 30 minutes. **b,** 35 wt. % paraffin wax @ 5 wt. % Ru/ activated carbon at absorbed power of ca. 80 W for a period of 32 minutes. **c,** 35 wt. % paraffin wax @ 5 wt. % Ru/ activated carbon at 20-15 W for a period of 90 minutes.

**Supplementary Fig. S2**

**Thermogravimetric analysis, b, the first derivative of the weight losses and c, differential scanning calorimetry for 50 wt. % paraffin wax @ activated carbon and 50 wt. % paraffin wax @ 5 wt. % Ru/activated carbon before and after the microwave-assisted catalytic decomposition of waxes.** The tests were made by irradiating the samples with 20-15 W for nearly 30 s, then the power was continuously increased to 30 W and held for another 30 s. The power was subsequently increased up to 60 W and held for 60 s. Finally, the delivered power was increased to 170-180 W and held for a period (30 min) until no further gas generation was detected.

**Supplementary Fig. S3**

**Comparisons of microwave and thermal treatment on the resulting evolved gas mixture composition**

**Supplementary Fig. S4**

**High-resolution phase-contrast HRTEM images of typical activated carbon-supported 5 wt. % ruthenium nanoparticles without paraffin wax impregnation.**

**Supplementary Table S1.**

**Maximum temperatures, weight losses and overall weight losses for 50 wt. % paraffin waxes @ activated carbon and 50 wt. % paraffin waxes @ 5 wt. % Ru/activated carbon before and after the microwave-assisted catalytic decomposition of waxes.**

**Supplementary Table S2.**

**Comparisons of the catalytic performances of microwave - activated carbon -supported noble metal catalysts blended with paraffin wax.**

**^a.^**

m_o(Cat)_ and m_f(Cat)_ are the weight of the catalyst (Cat) before and after the catalytic reaction, respectively. m_i_ is the weight of the gases at standard conditions for temperature and pressure (273 K; 100 kPa) and the liquid product and m_o_ corresponds to the initial weight of the paraffin waxes.

**^b.^** Paraffin wax (PW) conversion corresponds to the amount of transformed wax upon the microwave treatment.

where m_o_ or m_f_ corresponds to the initial or final weight of the paraffin waxes, respectively.

**^c.^**

where P_i_ is product(s) i (i.e. H_2_, CH_4_, liquids, C_2_-C_4_^=^ and C_2_-C_5_), m_i_ is the weight of the gases at standard conditions for temperature and pressure (273 K; 100 kPa) or the corresponding liquid product.

**Supplementary Table S3.**

**Influence of the heating sources (microwave vs conventional thermal furnace) over the catalytic decomposition of paraffin wax.** The microwave-assisted experiment was carried out at 80 W for 30 minutes of reaction time, whilst the thermally treated experiments were made in a furnace set at 823 K for 20 minutes until no further gas generation was detected.

**Supplementary Table S4.**

**The d-spacings of pure ruthenium metal (space group P6_3_/mmc) along with the values measured from the diffractograms in Fig. 4E for 58 wt. % of paraffin wax @ 5 wt. % Ru/AC after the microwave-assisted catalytic decomposition of paraffin wax. The error bar for the sampling in reciprocal space is 0.04 nm^-1^.**

**Supplementary Discussion**

The evolution of the reaction rate for the production of hydrogen and methane as major gases generated upon the microwave-assisted catalytic decomposition of paraffin waxes with the reaction time is illustrated in Supplementary Fig. S1. The hydrogen formation rates are clearly higher than those associated with the methane production whatever the presence (or absent) of metal catalyst and the adsorbed power. Note that the presence of Ru functionality enhances markedly the hydrogen production rate compared to the Ru-free formulation (Supplementary Figs. S1a and S1b) at similar adsorbed power. Furthermore, the diminution of the absorbed power to 20-15 W markedly decrease the production rates of hydrogen and methane despite the presence of Ru catalyst (Supplementary Fig. S1c).

We investigated a series of representative samples by both thermogravimetric analysis (TGA) and differential scanning calorimetry (DSC) under oxygen-free nitrogen flow to determine the influence of the activated carbon and 5 wt. % Ru on the activated carbon on the pyrolytic degradation of paraffin waxes. Two major weight losses are displayed in Supplementary Fig. S2a. The first one between 450 and 625 K with a maximum rate of weight loss change at 575 K (Supplementary Fig. S2b) is clearly associated to the evaporation of paraffin waxes since nearly 100 wt. % of this material is lost upon thermal treatment in the pure paraffin waxes TGA profile. The second weight loss between 650 and 825 K with a maximum derivative of weight loss at 768 K is tentatively attributed to the degradation (or evaporation) of paraffinic-type carbonaceous material (C_x_H_y_) produced by the interaction of activated carbon with melted paraffin waxes. Note that this last weight loss markedly decreased in the spent 50 wt. % paraffin wax @ 5 wt. % Ru/AC in sharp contrast to the Ru-free sample, suggesting that the Ru metal catalyses the decomposition of this refractory paraffinic-derived material. The DSC profiles for these samples show not only the pertinent endothermic peaks associated to these major weight losses (or decomposition processes) but also a strong endothermic peak at 331 K attributed to the melting process of the paraffin waxes (Supplementary Fig. S2c), since it is not observed any weight loss in this step. The spent samples did not show evident peak(s) associated to residual paraffin wax whilst the activated carbons showed a weak endothermic peak at 335 K because of moisture loss.

The gas composition of the 50 wt. % paraffin wax @ 5 wt. % Ru/AC under dielectric heating and conventional thermal heating also revel a slight increase of the hydrogen production and reduction of methane in the sample treated under microwave irradiation relative to the equivalent composite treated in a furnace at 823 K. It is worth remarking that the average temperature for the system under dielectric heating (ca. 723 K) was indeed significantly lower than that used in the thermal-treated sample (823 K). This finding highlights the remarkable effect of the selective heating of the microwave radiation and hence the dual effect of the hot and cold spots over the overall gas distribution. Note that Ru catalyst also enhanced the hydrogen production and decreased the methane formation compared to the non-metal-containing composite (50 wt. % paraffin wax @ AC) under conventional thermal heating.

The average crystal size of the Ru catalyst on the carbon support without paraffin wax is below 3 nm. The crystal shaper reflects a projected hexagon. Typical examples are shown in Supplementary Figs. S3a and S3b.

The series of experiments given in Supplementary Table S2 was carried out by irradiating the samples with an absorbed microwave power of 20-15 W for nearly 30 s, then the power was continuously increased to 30 W and held for another 30 s. Subsequently, the power was increased up to 60 W and held for 60 s. Finally, the delivered power was increased to 170-180 W and held for an extended period (i.e. 20-35 min) until no further gas generation was detected. The results given in Supplementary Table S2 represent the various product yields at the end of this sequence.

Based on the hydrogen production and the chemical composition of the major component of the paraffin wax (i.e. C_26_H_54_), we should expect a relatively low carbon yield from the 50.0 wt. % PW@AC formulation (i.e. 9.75 wt. %), Supplementary Table S2. This would indicate that the remaining yield (ca. 45 wt. %) corresponds mainly to residual carbonaceous material (C_x_H_y_), as was confirmed by thermo-gravimetric analysis (see Supplementary Figs. S2A, S2B and Table S1), since a relatively low yield of carbon oxides (CO+CO_2_) was obtained (i.e. 2.5 wt. %). Note that the generation of CO_x_ is mainly associated to the decomposition of –COO and –CO groups present in activated carbons. We should also expect significantly higher carbon yields for noble metal-containing formulations (ca. 35 -45 wt. %) because of the larger production of hydrogen. A mild re-oxidation of metal catalysts is also envisaged.

The liquid yield for metal particle catalysts, that is, hydrocarbons having a range of composition between C_7_ and C_17_, slightly increased compared to that produced by the 50 wt. % paraffin wax @AC sample.

In Supplementary Table 3S, the mass balance, the PW conversion and the H_2_ yield for 50 wt. % PW@5 wt. % Ru/AC are higher, while the methane and C_2_-C_5_ yields are lower than those obtained in the equivalent sample thermally treated in a furnace at 823 K. There is also a positive effect of the 5 wt. % Ru/AC catalyst over the PW conversion and hydrogen yield compared to the Ru-free sample (i.e. 50 wt. % PW@AC).

A relatively low mass balance for the thermally treated samples is most likely due to the formation of liquid hydrocarbons with low boiling points (C_5_-C_6_) that were not trapped in the cold trap (Fig. 3). These findings reveal the favourable, important effect of the microwave radiation to effectively release hydrogen from the paraffin wax despite the relatively low surface temperature of the catalyst bed (i.e. 573-773K) during the irradiation time.

**Supplementary Video**

Supplementary Video 1 (Edwards _supplementary_Video.mp4)

<https://www.dropbox.com/s/zzttvbhgidclngs/Movie%20S1.mp4?dl=0>

**Supplementary Video Legend**

Video illustrating the rapid production of hydrogen from microwave-assisted decomposition of paraffin wax. The catalyst-wax mixture was exposed to a uniform microwave electric field at a frequency of 2.45 GHz, with an absorbed power of 150W. Hydrogen is generated almost instantaneously as the microwaves are cycled on and off.
